# Photonic transistor and router using a single quantum-dot-confined spin in a single-sided optical microcavity

**DOI:** 10.1038/srep45582

**Published:** 2017-03-28

**Authors:** C. Y. Hu

**Affiliations:** 1 Department of Electrical and Electronic Engineering, Merchant Venturers School of Engineering, Faculty of Engineering, University of Bristol, Woodland Road, Bristol, BS8 1UB, United Kingdom

## Abstract

The future Internet is very likely the mixture of all-optical Internet with low power consumption and quantum Internet with absolute security guaranteed by the laws of quantum mechanics. Photons would be used for processing, routing and com-munication of data, and photonic transistor using a weak light to control a strong light is the core component as an optical analogue to the electronic transistor that forms the basis of modern electronics. In sharp contrast to previous all-optical tran-sistors which are all based on optical nonlinearities, here I introduce a novel design for a high-gain and high-speed (up to terahertz) photonic transistor and its counterpart in the quantum limit, i.e., single-photon transistor based on a linear optical effect: giant Faraday rotation induced by a single electronic spin in a single-sided optical microcavity. A single-photon or classical optical pulse as the gate sets the spin state via projective measurement and controls the polarization of a strong light to open/block the photonic channel. Due to the duality as quantum gate for quantum information processing and transistor for optical information processing, this versatile spin-cavity quantum transistor provides a solid-state platform ideal for all-optical networks and quantum networks.

Big data, cloud computing and rapidly increasing demand for speed or bandwidth are driving the new generation of Internet which is intelligent, programmable, energy-efficient and secure. The current Internet is not fully transparent and continues to deploy electronic information processing with energy-consuming optical-electrical/electrical-optical conversions^1^. The long-sought technology for optical information processing (OIP) (or photonic transistor (PT))^2^ and optical buffering^3^ are not around the corner yet, and hinders the development of all-optical networks. Moreover, the current Internet is not very secure as it uses light pulses to transmit information across fiber-optic networks. These classical optical pulses can be easily intercepted and copied by a third party without any alert. Quantum Internet^4^ uses individual quanta of light, i.e., photons to encode and transmit information. Photons can not be measured without being destroyed due to the no-cloning theorem in quantum mechanics^5^, so any kind of hacking can be monitored and evaded. The realization of quantum Internet requires not only quantum gates and quantum memories for quantum information processing (QIP), but also single-photon transistor (SPT) gated by a single photon to control the flow of information.

The future Internet is very likely the mixture of all-optical Internet with low power consumption and quantum Internet with absolute security. The optical regular Internet would be used by default, but switched over to quantum Internet when sensitive data need to be transmitted. PT and and its counterpart in the quantum limit SPT would be the core components for both OIP and QIP in future Internet. Compared with electronic transistors, PTs/SPTs potentially have higher speed, lower power consumption and compatibility with fibre-optic communication systems.

Several schemes for PT^6^,^7^,^8^,^9^,^10^ and SPT^11^,^12^,^13^,^14^,^15^,^16^,^17^ have been proposed or even proof-of-principle demonstrated. All these prototypes exploit optical nonlinearities, i.e., photon-photon interactions^18^. However, photons do not interact with each other intrinsically, so indirect photon-photon interactions via electromagnetically induced transparency (EIT)^19^, photon blockade^20^ and Rydberg blockade^21^ were intensively investigated in this context over last two decades in either natural atoms^22^,^23^ or artificial atoms including superconducting boxes^24^,^25^ and semiconductor quantum dots (QDs)^12^,^13^. PT can seldom work in the quantum limit as SPT with the gain greater than 1 because of two big challenges, i.e., the difficulty to achieve the optical nonlinearities at single-photon levels and the distortion of single-photon pulse shape and inevitable noise produced by these nonlinearities^26^. The QD-cavity QED system is a promising solid-state platform for information and communication technology (ICT) due to their inherent scalability and matured semiconductor technology. But the photon blockade resulting from the anharmonicity of Jaynes-Cummings energy ladder^27^ is hard to achieve due to the small ratio of the QD-cavity coupling strength to the system dissipation rates^12^,^13^,^28^,^29^,^30^,^31^,^32^ and the strong QD saturation^33^. Moreover, the gain of this type of SPT based on the photon blockade is quite limited and only 2.2 is expected for In(Ga)As QDs^12^,^13^.

In this work, a different PT and SPT scheme exploiting photon-spin interactions rather than photon-photon interactions is proposed based on a linear quantum-optical effect - giant optical Faraday rotation (GFR) induced by a single QD-confined spin in a single-sided optical microcavity^34^. This spin-cavity transistor is genuinely a quantum transistor in three aspects: (1) it is based on a quantum effect, i.e., the linear GFR; (2) it has the duality as a quantum gate for QIP and a classical transistor for OIP; (3) it can work in the quantum limit as a SPT to amplify a single-photon state to Schrödinger cat state. Therefore this new-concept transistor can be more powerful than the traditional electronic transistors. Theoretically the maximum gain can reach ~10^5^ in the state-of-the-art pillar microcavity, several orders of magnitude greater than previous PT/SPT schemes^6^,^7^,^8^,^9^,^10^,^11^,^12^,^13^,^14^,^15^,^16^,^17^. The large gain is attributed to the linear GFR that is robust against classical and quantum fluctuations and the long spin coherence time compared with the cavity lifetime. The maximal speed which is determined by the cavity lifetime has the potential to break the terahertz (THz) barrier for electronic transistors^35^,^36^. Based on this versatile spin-cavity transistor, optical Internet^1^, quantum computers (QCs)^37^,^38^ (either spin-cavity hybrid QCs or all-optical QCs), and quantum Internet^4^ could become reality even with current semiconductor technology.

## Results and Discussions

### Linear GFR for robust and scalable quantum gates

A single electron (or hole) spin confined in a charged QD in an optical microcavity can induce GFR^34^ – a kind of optical gyrotropy (or optical activity)^39^. Although cavity QED systems are typically highly nonlinear, it was recently found that GFR exhibits both nonlinear and linear behaviors^33^. The nonlinear GFR is sensitive to the power of incoming light occurs when the QD is partially saturate, and has been demonstrated experimentally for single QD spin^40^,^41^,^42^,^43^,^44^, QD spin ensemble^45^, and single atom^46^,^47^,^48^ in the weak coupling regime of cavity QED or without a cavity. The linear GFR is independent of the power of incoming light and occurs when the QD is pinned to the ground state within the non-saturation window (NSW) or in the weak-excitation limit. The linear phase shift was reported recently in strongly-coupled atom-cavity systems^49^,^50^. The linear GFR in QD-cavity systems is responsible for both robust quantum gate operations (in this subsection) and transistor operations (in the next subsection) although it has not been demonstrated yet.

**Figure 1:**
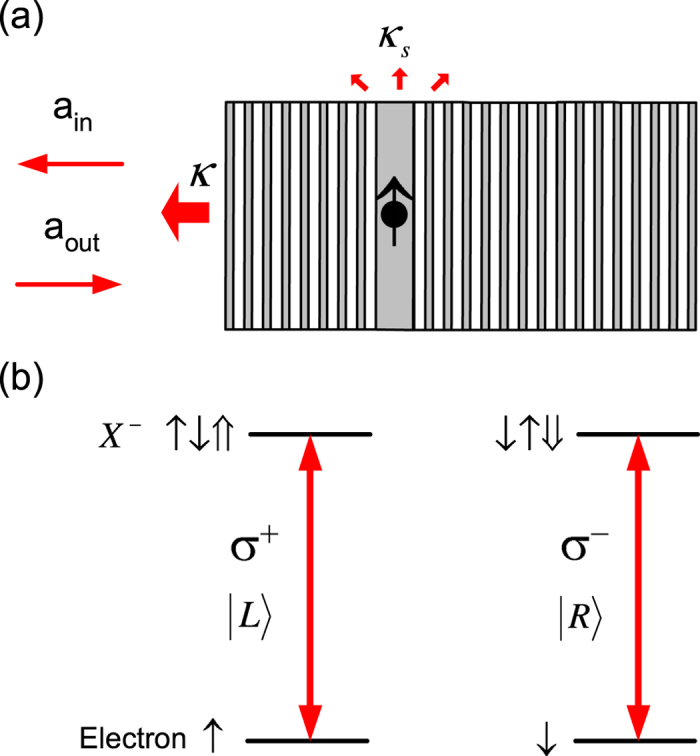
Structure of the spin-cavity unit. (**a**) A charged quantum dot is embedded in a single-sided pillar microcavity with one end mirror partially reflective and another end mirror 100% reflective allowing unit cavity reflectance when the cavity side leakage is neglected. The circular pillar cross-section supports circularly polarized light. (**b**) Optical transitions in a negatively-charged quantum dot follow spin selection rules: a photon in the |*L*〉 state couples to the transition |↑〉 ↔ |↑↓⇑〉 only, whereas a photon in the |*R*〉 state couples to the transition |↓〉 ↔ |↓↑⇓〉 only due to the conservation of angular momentum and the Pauli exclusion principle.

Figure 1(a) shows such a spin-cavity unit with a negatively charged QD in a single-sided pillar microcavity. This type of cavity can be fabricated from a planar microcavity defined by two distributed Bragg reflectors (DBRs) with the cavity length chosen to be one wavelength *λ* such that the field maxima is situated in the middle of the planar microcavity. The front mirror is made partially reflective and the back mirror 100% reflective, allowing unit cavity reflection if the cavity side leakage is negligibly small. Three-dimensional confinement of light is provided by the two DBRs and the transverse index guiding. The cross section of the micropillar is made circular in order to support circularly polarized light besides linearly polarized light. Some photonic crystal nanocavities with specific spatial symmetry^51^ could support circularly polarized light and are suitable for this work, too. However, the advantage to use the pillar microcavity is the high coupling efficiency as the fundamental cavity mode which is gaussian-like can match perfectly with the external laser beam.

A negatively (or positively) charged QD has an excess electron (or hole) confined in the QD. Charging a QD can be achieved via modulation doping, tunneling^52^, or optical injection. The ground states of the charged QD are the electron (or hole) spin states, and the excited states are the spin states of the negatively charged exciton *X*^−^ (or positively charged exciton *X*^+^) [see Fig. 1(b)]. In the absence of external magnetic field, both the ground and excited states of charged QD are two-fold degenerate due to the Kramers theorem. The electron spin degeneracy could be lifted by the nuclear spin magnetic fields^53^ via the electron-nucleus hyperfine interactions in In(Ga)As QDs, however, the Zeeman splitting is too small to spoil the linear GFR^33^. The hole spin degeneracy is not affected by the nuclear spin fields due to the lack of the hole-nucleus hyperfine interactions.

Due to the conservation of total spin angular momentum and the Pauli exclusion principle, the left circularly polarized photon (marked by |*L*〉 or |*σ*^+^〉) only couples to the transition |↑〉 ↔ |↑↓⇑〉, and the right circularly polarized photon (marked by |*R*〉 or |*σ*^−^〉) only couples to the transition |↓〉 ↔ |↓↑⇓〉 [see Fig. 1(b)]. Here |↑〉 and |↓〉 represent electron spin states 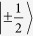, |⇑〉 and |⇓〉 represent heavy-hole spin states 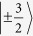 with the spin quantization axis z along QD growth direction, i.e., the input/output direction of light. The weak cross transitions due to the heavy-hole-light-hole mixing^54^ can be corrected and are neglected in this work. Note that the photon polarizations are marked by the input states to avoid any confusion due to the temporary polarization changes upon reflection.

If the spin is in the |↑〉 state, a photon in the |*L*〉 state can couple to the QD and feels a “hot” cavity, whereas a photon in the |*R*〉 state can not couple to the QD and feels a “cold” cavity [see Fig. 1(b)]. If the spin is in the |↓〉 state, a |*R*〉-photon feels a “hot” cavity and a |*L*〉-photon feels a “cold” cavity.

Although the incoming light is fully reflected back from a single-sided cavity, there exists significant phase difference between the reflection coefficients of the “hot” and “cold” cavity as the QD-cavity interactions can strongly modify the cavity properties. This cavity-QED effect is verified by the calculations using two approaches (see Methods): an analytical method by solving Heisenberg-Langevin equations of motions in the semi-classical approximation, and a numerical but exact method by solving master equation with a quantum optics toolbox^55^,^56^. The calculated results using these approaches can be found in our previous work^33^.

The phase difference between the “hot” and “cold” cavity can be mapped to that between left-handed and right-handed circularly polarized light. This results in the optical rotation of polarization of a linearly polarized light after reflection, i.e., GFR effect which is induced by a single spin. GFR can be regarded as a macroscopic imprint of the microscopic spin selection rules for optical transitions in charged QD (sometimes it is also called Pauli blockade) [see Fig. 1(b)]. GFR is a type of magnetic optical gyrotropy (also known as magnetic optical activity) in the presence of magnetic field or magnetization^39^. A key feature of GFR is its spin tunability, which makes the spin-cavity unit versatile for QIP and OIP. Another merit is the linearity of GFR that remains constant with increasing the power of incoming light.

The linear GFR occurs around the cavity resonance in the strong coupling regime g ≫ (*κ* + *κ*_*s*_,*γ*) or in the Purcell regime *γ* < 4g^2^/(*κ* + *κ*_*s*_) < (*κ* + *κ*_*s*_) when the input power is less than *P*_*max*_ (see Methods for its definition) such that the QD stays in the ground state. Within the NSW or in the weak-excitation limit, the coherent scattering dominates the reflection process and the semi-classical approximation yields the same results as the full quantum model^33^. Taking 〈*σ*_*z*_〉 = −1, the steady-state reflection coefficient can be obtained from Eq. (12) in Methods,


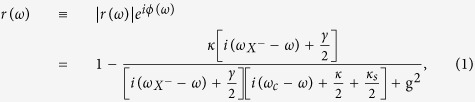


where *ω, ω*_*c*_, 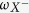 are the frequencies of incoming light, cavity mode, and the *X*^−^ transition, respectively. g is the QD-cavity coupling strength which is given by 
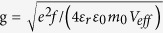
 (if the QD is placed at the maxima of cavity field) with *f* being the *X*^−^ oscillator strength (*f*:10–100 for InAs- or GaAs-based QDs) and *V*_*eff*_ being the cavity mode volume. *κ*/2 is the the cavity field decay rate into the input/output port, and *κ*_*s*_/2 is the side leakage rate of the cavity field including the material background absorption. *γ*/2 is the total QD dipole decay rate including the spontaneous emission rate 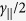 into leaky modes and the pure dephasing rate γ^*^, i.e., 
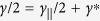
. The pure dephasing rate can be neglected when the QD stays in the ground state as being proved in recent experiments on generation of high-quality single photons from In(Ga)As QDs under weak resonant excitation^57^,^58^. For pillar microcavity, the spontaneous emission rate into leaky modes is approximately equal to the free-space emission rate as the reduced density of leaky modes can be compensated by the Purcell enhancement due to the light confinement from the planar cavity.

For convenience to discussions, the resonant condition 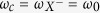 is assumed in this work although this assumption is not necessary as the linear GFR is tolerable to the frequency mismatch 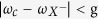. In the one-dimensional atom regime where 4g^2^ ≫ (*κ* + *κ*_*s*_)*γ* (this includes the strong coupling regime and part of the Purcell regime), Eq. (1) yields 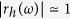 and 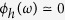 when the light frequency *ω* lies within the NSW, i.e., |*ω* − *ω*_0_| < −*g*〈*σ*_*z*_〉 where 〈*σ*_*z*_〉 depends on the intensity of the incoming light. If the cavity side leakage is smaller than the input/output coupling rate, i.e., *κ*_*s*_ ≪ *κ*, 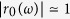 and *ϕ*_0_(*ω*) ∈ [−*π*, +*π*] can be achieved for the cold cavity. The spin-dependent (conditional) phase shift can be used to build a deterministic photon-spin entangling gate (precisely speaking, a conditional phase gate or a parity-check gate) with the phase shift operator defined as ref. 34


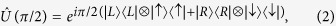


where the phase shift Δ*ϕ* = *ϕ*_*h*_ − *ϕ*_0_ is chosen to ±*π*/2 by setting the frequency detuning Δ*ω* = *ω* − *ω*_0_ ≈ ±(*κ* + *κ*_*s*_)/2. Higher gate fidelity can be achieved in strongly coupled QD cavity QED systems which has been demonstrated in various micro- or nano-cavities^12^,^13^,^28^,^29^,^30^,^31^,^32^. In the state-of-the-art pillar microcavities^28^,^59^, g/(*κ* + *κ*_*s*_) = 2.4 is achieved for In(Ga)As QDs and this parameters is used for judging the performance of transistor in this work.

As the linear GFR occurs when the QD stays in the ground state, this photon-spin entangling gate is robust against quantum fluctuations^33^ either from the inside of cavity or from the outside, e.g., the intensity fluctuations of the incoming light. The linear GFR within the NSW is also resistant to spectral diffusion, pure dephasing, charge and nuclear spin noise, high-order dressed state resonances (i.e., the higher manifolds of the Jaynes-Cummings ladder), and small QD-cavity mismatch 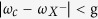 which could be induced by external electric/magnetic fields, all of which could occur in realistic QDs. This universal, deterministic and robust photon-spin entangling gate is promising for scalable solid-state QIP.

It is worth noting that rather than the *π*/2 phase shift used here, the conditional *π* phase shift can be also used for a controlled phase flip gate in a strongly coupled atom-cavity system^60^. Since the pioneer work by Kimble’s group^46^, both nonlinear^47^,^48^ and linear^49^,^50^ phase shift of *π* (and *π*/2 by frequency detuning) have been demonstrated in atomic cavity-QED systems recently. Significant progress has also been made in QD-cavity systems, e.g., the nonlinear GFR of ~6° induced by a single hole spin^43^ or electron spin^44^, a photon sorter^61^, and a quantum phase switch^62^. However, all these experiments were performed in the weak coupling regime of cavity QED^63^,^64^ (equivalent to waveguide-QED^65^) where the nonlinear GFR (or the nonlinear phase shift) dominates and the GFR bandwidth is quite small (limited by the QD linewidth). The linear GFR in strongly-coupled QD-cavity systems is desired as it occurs with a broader bandwidth (limited by the cavity mode linewidth) and allows both robust quantum gate and transistor operations.

For the sake of convenience, some properties of the photon-spin phase shift operator 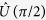 are listed below


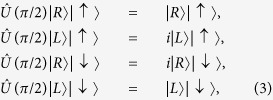


and


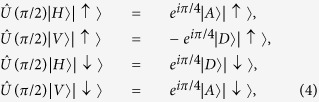


where 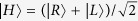 and 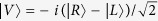 are linear polarization states, 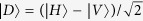 and 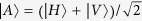 are diagonal linear polarization states. It is worth noting that |*R*〉|↑〉, |*L*〉|↑〉, |*R*〉|↓〉, and |*L*〉|↓〉 are eigen states of the spin-cavity hybrid device. The linearly polarized light will turn its polarization by ±45° after reflection (i.e., GFR effect) if the spin is set to |↑〉 or |↓〉. These properties are used in this work.

**Figure 2:**
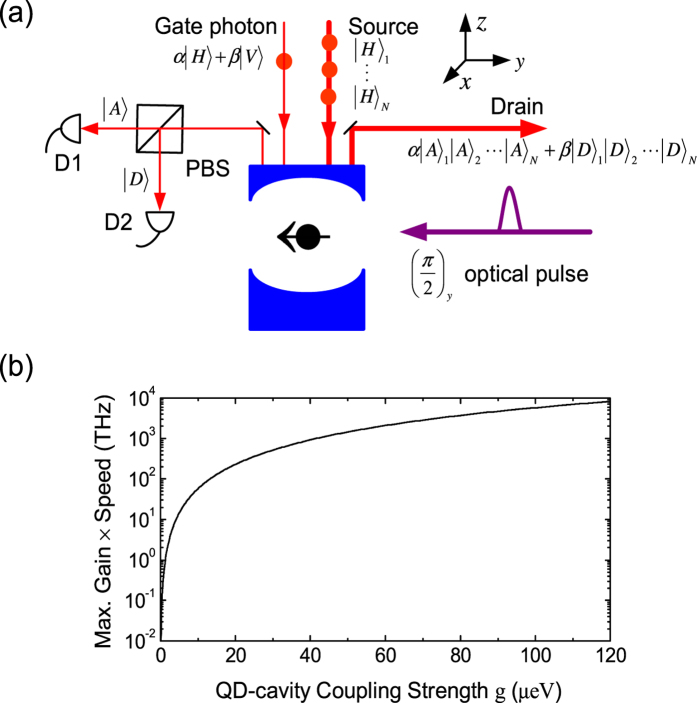
Diagram of the spin-based photonic transistor. (**a**) Firstly, the spin is initialized to 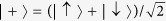, and the gate photon is prepared in an arbitrary state |*ψ*^*ph*^〉 = *α*|*H*〉 + *β*|*V*〉; Secondly, the gate photon state is transferred to spin by measuring the photon in the {|*D*〉, |*A*〉} basis after reflection; Thirdly, the spin state is transferred to N photons injected from the source S by measuring the spin in the {|+〉, |−〉} basis. As a result, an arbitrary quantum state of a gate photon is transferred (or “amplified”) to the same state encoded on N photons in the channel. PBS (polarization beam splitter), D1-D2 (single photon detectors). Note that the PBS is oriented in the {|*D*〉, |*A*〉} basis, and transmits the photon in state |*A*〉 and reflects the photon in state |*D*〉. (**b**) The maximum gain – speed product as a function of the QD-cavity coupling strength g. The spin coherence time is taken as *T*_2_ = 1 *μ*s (see the text).

By itself the spin-cavity unit can be used to initialize the spin via single-photon based spin projective measurement together with classical optical pulses injected from the cavity side. Assume the spin is in a unknown state *α*|↑〉 + *β*|↓〉, and the incoming photon in the |*H*〉 state. After the photon-spin interaction, the photon and spin become entangled, i.e., *α*|*A*〉|↑〉 + *β*|*D*〉|↓〉, On detecting the photon in |*A*〉, the spin is projected to |↑〉. On detecting the photon in |*D*〉, the spin is projected to |↓〉. To convert the spin from |↓〉 to |↑〉 or vice versa, a spin rotation of *π* around *y* axis is required (see Fig. 2(a) for the definition of *x, y, z* axes). This can be achieved using a ps or fs optical (*π*)_*y*_ pulse (injected from the cavity side^66^) via the optical Stark effect^67^. To prepare the superposition state such as 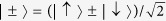, an optical 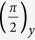 pulse can be applied. With these techniques, the electron spin in a pillar microcavity can be prepared to an arbitrary state deterministically.

Spin coherence time *T*_2_ is an important parameter for both quantum gate and transistor operations. In GaAs-based or InAs-based QDs, the electron spin dephasing time 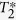 can be quite short (~ns) due to the hyperfine interaction between the electron spin and 10^4^ to 10^5^ host nuclear spins^53^. To suppress the nuclear spin fluctuations, spin echo or dynamical decoupling techniques^68^ could be applied to recover the electron spin coherence using optical pulses^67^ and/or single-photon pulses^69^. Based on the spin echo techniques, the electron spin coherence time *T*_2_ = 1*μ*s has been reported recently in a single In(Ga)As QD^67^, which is taken in this work to estimate the transistor gain-speed product (see Fig. 2(b)). Note that the spin echo technique is compatible with the quantum gate and transistor operations in the spin-cavity unit.

Aside from the deterministic photon-spin entangling gate, this spin-cavity unit can also work as deterministic photon-photon, spin-spin entangling gates and deterministic photon-spin interface or heralded spin memory, single-shot quantum non-demolition (QND) measurement of single spin or photon, complete Bell-state generation, measurement and analysis as well as on-chip quantum repeaters^34^,^69^,^70^,^71^,^72^. Assisted by single photon or single spin gate operations, these gates can be converted to the popular quantum gates in different systems, e.g., controlled-NOT (CNOT) or hyper CNOT gates, Toffoli gates, and Fredkin gates which can be useful for scalable quantum computing^73^,^74^,^75^,^76^. Similar quantum gates, i.e., the controlled phase-flip gates were proposed by Duan and Kimble^60^ in atom-cavity systems have been experimentally demonstrated recently^49^,^50^. However, the atomic systems require complicated and expensive equipment for cooling and trapping and the scalability is really a big challenge from a practical point of view. Moreover, in atomic systems these quantum gates work in the MHz range due to the small atom-cavity coupling strength. The QD-cavity systems have inherent scalability, high speed (tens to hundreds of GHz) due to the larger QD-cavity coupling strength, and the ease to fabricate with matured semiconductor technology, thus providing an ideal platform for solid-state QIP.

### Linear GFR for photonic transistor

The spin-dependent linear GFR can be utilized to make a spin-based SPT as shown in Fig. 2(a). A gate photon sets the spin state via projective measurement and controls the polarization of light in the photonic channel between the source S and the drain D.

The spin is initialized to 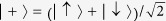 using single-photon based spin projective measurement in combination with an ultrafast optical pulse injected from the cavity side. The gate photon is prepared in an arbitrary state |*ψ*^*ph*^〉 = *α*|*H*〉 + *β*|*V*〉. After the photon interacting with the spin, the joint state becomes


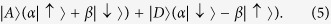


The photon is then measured in the {|*D*〉, |*A*〉} basis. On detecting the photon in |*A*〉 (a click on D1), the spin is projected to |*ψ*^*s*^〉 = *α*|↑〉 + *β*|↓〉. On detecting the photon in |*D*〉 (a click on D2), the spin is projected to |*ψ*^*s*^〉 = *α*|↓〉 − *β*|↑〉, which can be converted to |*ψ*^*s*^〉 = *α*|↑〉 + *β*|↓〉 by spin rotations. The spin-cavity unit works as a photon-spin quantum interface, with which the photon state is transferred to spin state deterministically.

Assume there are N photons in the |*H*〉 state from the source S (the photon number N is the maximum gain which will be discussed later). After all photons interacting with the spin in sequence, the joint state becomes


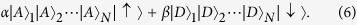


An optical 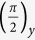 pulse is then applied from the cavity side to perform the spin Hadamard transformation. After that, a photon in the |*H*〉 state is sent to perform the spin measurement, and the joint state are projected to a superposition state


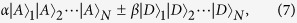


where “+” is taken for spin |+〉 and “−” for spin |−〉. The negative sign can be converted to the positive using a phase shifter.

As a result, an arbitrary quantum state of a single gate photon is transferred or “amplified” to the same state encoded on N photons, which is of Greenberger-Horne-Zeilinger (GHZ) state like or Schrödinger-cat state like^77^. In this sense, this SPT is genuinely a quantum transistor based on a quantum effect – GFR, and exhibits the dual nature as a quantum gate and a transistor. As the original state of the gate photon is destroyed after the transistor operation, this SPT does not violate the no-cloning theorem in quantum mechanics^5^. This transistor could generate entanglement of hundreds to thousands of photons and has the potential to break the current record of 10-photon entanglement^78^. The multiphoton entanglement are essential to quantum communications^79^ and quantum metrology^80^. Previous calculations of the entanglement fidelity and efficiency in terms of single-photon transportation^34^,^70^ can be extended to the case of n-photon Fock state (*n* ≤ *P*_*max*_) as long as the linear GFR preserves when the incoming light power is less than *P*_*max*_. This is because the n-photon transitions for the excitation of the n^th^ manifold of dressed states do not affect the linear GFR occurring within the NSW, and the reflection amplitude and phase remain the same for both single-photon and n-photon Fock states^33^. The efficiency and fidelity to generate the N-photon GHZ- or cat-like states in Eq. (7) depends on cavity QED parameters (*g, κ, κ*_*s*_, *γ*), the spin coherence time *T*_2_, the time interval between photons as well as the precision for spin manipulations^69^.

The time interval between the channel and gate photons should be less than by the spin coherence time *T*_2_ which defines the time window for the transistor operation. The photon rate in the channel can go as high as *P*_*max*_ where the linear GFR preserves. As the spin state can be controlled by a single photon, the maximum gain of the SPT is exactly the maximum photon number allowed in the channel, i.e.,


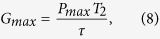


where *τ* is the cavity lifetime which determines the cutoff frequency *f*_T_ of SPT (i.e., the maximum speed). The maximum gain-speed product is thus


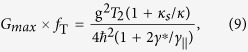


where *ħ* is the reduced Planck constant. The gain-speed product increases with increasing the coupling strength g or the spin coherence time *T*_2_ [Fig. 2(b)]. In the state-of-the-art pillar microcavity^28^,^59^ where g/(*κ* + *κ*_*s*_) = 2.4 (g = 80 *μ*eV, *κ* + *κ*_*s*_ = 33 *μ*eV), the maximum gain can reach 7 × 10^4^, which surpasses all other SPT protocols by several orders of magnitude^11^,^12^,^13^,^14^,^15^,^17^. However, the high gain is at the cost of a low speed (*f*_T_ ~ 50 GHz in this case) and vice versa. In order to raise the speed, the cavity lifetime can be reduced. For example, if the cavity decay rate increases to 660 *μ*eV, *f*_T_ goes up to 1 THz with the gain down to 3.5 × 10^3^. However, too much cavity decaying will wash out GFR^34^ if 4g^2^/(*κ* + *κ*_*s*_) < *γ*, and causes the failure of the SPT operation.

Besides its quantum nature, the SPT can also work as a classical PT: a gate photon in a mixed state of |*H*〉 and |*V*〉 can be amplified to the same mixed state of N photons. Moreover, this classical PT also works if a gate photon is replaced by a classical optical pulse as long as the optical power is much less than *P*_*max*_. It is worth noting that this spin-cavity transistor satisfies most criteria for all-optical transistors that could compete with the electronic counterparts^2^, e.g., logic level independent of loss due to the linear GFR which is immune to the power variations of incoming light.

Different from the spin-based electronic transistors exploiting the Rashba spin-orbit interactions^81^, this spin-based PT exploiting the spin-cavity interactions does not suffer from the limitation from the RC time constants and the transit time, so it has the potential to break the THz barrier for all electronic transistors including the state-of-the-art high electron mobility transistors (HEMTs)^35^,^36^. Moreover, the PT consumes less energy as there exists neither Joule heating nor capacity charging in the photonic channel.

Similar to the conventional transistor, this spin-cavity transistor can be used as photonic switches or modulators with high-speed (up to THz). The inclusion of a spin into the cavity breaks the time inversion symmetry if the spin orientation is fixed. This results in the optical non-reciprocity^39^ which can be utilized for making various non-reciprocal photonic devices such as diodes, isolators and circulators. All these devices are useful for quantum networking and all-optical networking.

### Linear GFR for photonic router

In analogy to classical routers in regular Internet, which direct the data signal to its intended destination according to control information contained in the IP address, quantum routers^82^ are a key building block in quantum networks and quantum Internet, which direct a signal quantum bit (qubit) to its desired output port determined by the state of a control qubit, but keeping the state of the signal qubit unaltered.

**Figure 3:**
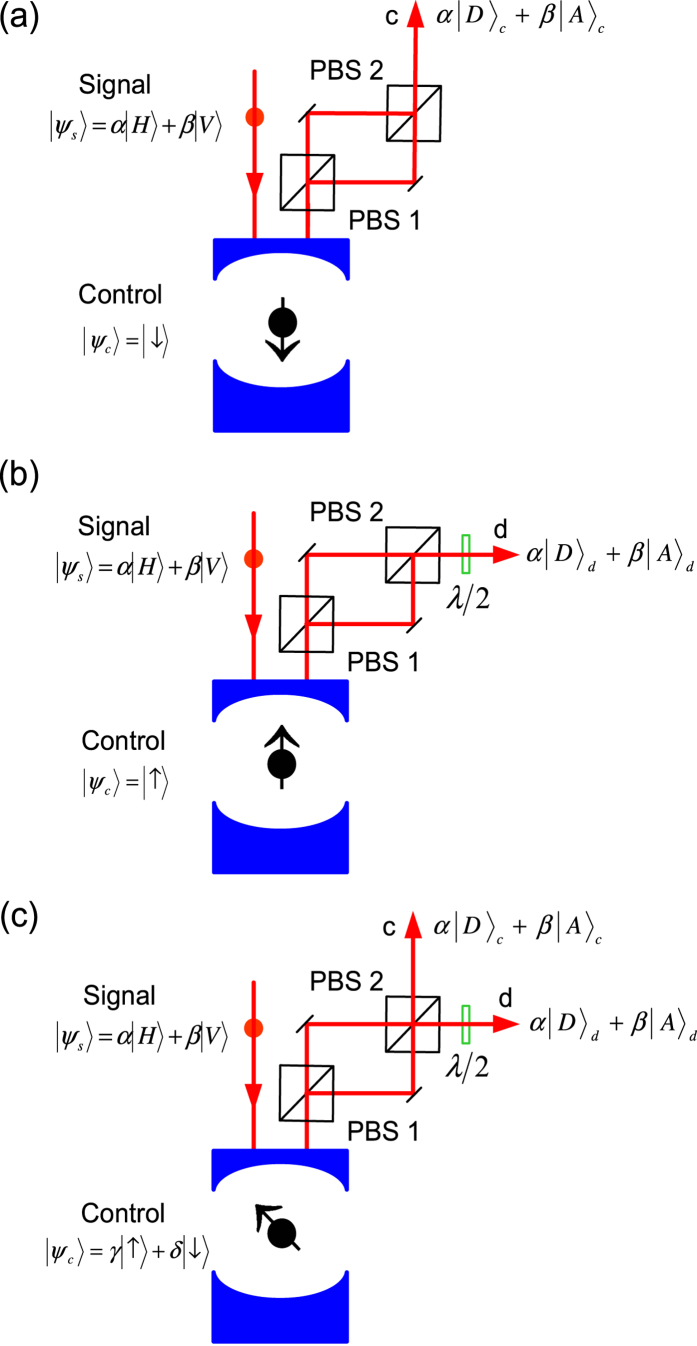
Diagram of the spin-based photonic router. The control spin directs the signal photon to: (**a**) port c; (**b**) port d; (**c**) superposition of two modes in port c and d. PBS1, PBS2 (polarization beam splitters), *λ*/2 (wave plate). Note that PBS1 is set to the {|*H*〉, |*V*〉} basis and PBS2 to the {|*D*〉, |*A*〉} basis.

The spin-cavity unit is an ideal component to make a quantum router (Fig. 3). The photon is used as the signal qubit encoded to an arbitrary state |*ψ*_*s*_〉 = *α*|*H*〉 + *β*|*V*〉 to be directed to its destination (port c or d), and the spin is used as the control qubit.

If the control spin is set to |*ψ*_*c*_〉 = |↓〉 [Fig. 3(a)], the signal photon state is changed from |*ψ*_*s*_〉 = *α*|*H*〉 + *β*|*V*〉 to |*ψ*_*s*_〉 = *α*|*D*〉 + *β*|*A*〉 after the photon-spin interaction. The photon state is then split by PBS1 and combined by PBS2, and finally the signal photon comes out from port c with its state unchanged, i.e, |*ψ*_*s*_〉 = *α*|*D*〉_*c*_ + *β*|*A*〉_*c*_.

If the control spin is set to |*ψ*_*c*_〉 = |↑〉 [Fig. 3(b)], the signal photon state is changed from |*ψ*_*s*_〉 = *α*|*H*〉 + *β*|*V*〉 to |*ψ*_*s*_〉 = *α*|*A*〉 − *β*|*D*〉 after the photon-spin interaction. After PBS1, PBS2, and a *λ*/2 wave plate, the signal photon comes out from port d with the state |*ψ*_*s*_〉 = *α*|*D*〉_*d*_ + *β*|*A*〉_*d*_, the same as the original one.

If the control spin is set to |*ψ*_*c*_〉 = *γ*|↑〉 + *δ*|↓〉, after the photon-spin interaction the joint output state becomes


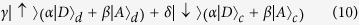


which is generally the superposition of two modes in port c and d [Fig. 3(c)]. Fully controlled by the spin state, the signal photon can be directed to port c, or port d, or the superposition of port c and d, whereas the signal photon state remains unchanged. As the spin-cavity unit can work as a deterministic photon-spin interface as discussed above, the control spin can be replaced by a photon, such that the quantum router becomes fully transparent. It is worthy noting that the PBS1 and PBS2 just split and recombine the polarization states, rather than forming an optical interferometer. Thus the phase instability is not an issue for this router as interference does not play a role here. Compared with another quantum router scheme based on linear optics^82^, this spin-cavity quantum router is deterministic and scalable to multiple-photon routing.

Aside from single-photon quantum router, the spin-cavity unit can also work as a classical photonic router if single photons are replaced by classical optical pulses as long as the light power is below *P*_*max*_. As the Joule heating and capacity charging are both absent in the photonic channel, photonic routers consume less energy and would replace the electronic routers in future energy-efficient Internet.

It is worth pointing out that the spin-cavity unit can also be used to route light (or photons) carrying orbital angular momentum (OAM)^83^ if OAM is converted to circular polarization of light or photons via a q-plate^84^.

## Conclusions and outlook

The spin-based high-gain and high-speed PT/SPT and related devices discussed above can be made in parallel based on giant circular birefringence (GCB) in another type of spin-cavity unit with a double-sided microcavity^85^,^86^. In both cases, the maximum transistor gain could reach ~10^5^ in the state-of-the-art pillar microcavity depending on the QD-cavity coupling strength and the spin coherence time. The maximal speed which is determined by the cavity lifetime could reach tens to hundreds of GHz and has the potential to break the THz barrier for electronic transistors.

An unusual feature of these spin-cavity units is the duality as quantum gates and transistors thanks to the linear GFR/GCB which are robust against quantum and classical fluctuations including the intensity variations of input light, spectral diffusion and pure dephasing in QDs, charge and spin noise in QDs, and even electric/magnetic fields. On the one hand, the spin-cavity units work as universal, deterministic and robust quantum gates for QIP. On the other hand, the spin-cavity units work as optical transistors for OIP. This work demonstrates that the spin-cavity units provide a solid-state platform ideal for future green and secure Internet - a combination of all-optical Internet^1^ with quantum Internet^4^, which is very likely to happen within the next 10–20 year timescale. This work series opens a new research field - spin photonics which studies the physics and applications of a single QD spin in different photonic structures including cavities, waveguides, chiral structures, and topological structures.

## Methods

### Semiclassical model

The Heisenberg equations of motions^87^ for the cavity field operator 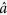 and the QD dipole operators *σ*_−_, *σ*_*z*_, together with the input-output relation^88^ can be written as


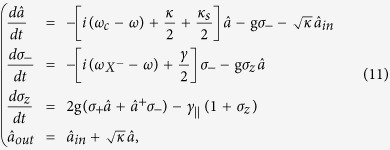


where all the parameters here have the same definitions and meanings as in Eq. (1).

If the correlations between the cavity field and the QD dipole are neglected (this is called the semiclassical approximation), 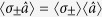 and 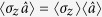. The semiclassical approximation can be applied in three cases: (1) low-power limit 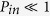 where the QD stays the ground state (weak-excitation approximation); (2) high-power limit 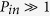 where the QD is saturated; (3) within the NSW where the QD is pinned to the ground state^33^. The reflection coefficient can thus be derived as


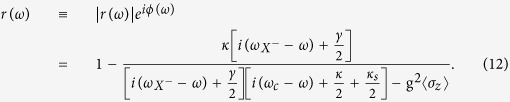


The population difference 〈*σ*_*z*_〉 is given by


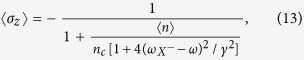


and the average cavity photon number 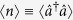 by


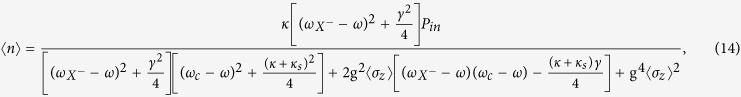


where 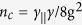 is the critical photon number which measures the average cavity photon number required to saturate the QD response, and *n*_*c*_ = 2.2 × 10^−4^ is taken in this work. 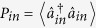 is the input light power. 〈*σ*_*z*_〉 is the QD population difference between the excited state and the ground state, and can be used to measure the saturation degree. 〈*σ*_*z*_〉 ranges from −1 to 0. If 〈*σ*_*z*_〉 = −1, the QD is in the ground state (not saturated); if 〈*σ*_*z*_〉 = 0, QD is fully saturated, i.e., 50% probability in the ground state and 50% probability in the excited state. If 〈*σ*_*z*_〉 takes other values, the QD is partially saturated.

By solving Eqs (13) and (14), 〈*σ*_*z*_〉 and 〈*n*〉 can be obtained at any input power. Note that 〈*σ*_*z*_〉 and 〈*n*〉 are dependent on the input power, frequency *ω* and coupling strength g. Putting 〈*σ*_*z*_〉 into Eq. (12), the reflection coefficient can be derived.

The linear GFR persists as long as the NSW between the first-order dressed state (or polariton state) resonances is open^33^. From Eqs (13) and (14), it can be estimated that the NSW is closed roughly at 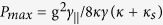 when 〈*σ*_*z*_〉 = −1/2. The higher the coupling strength g, the higher powers the linear GFR can preserve.

### Full quantum model - master equation

The reflection coefficient can be also calculated numerically in the frame of master equations in the Lindblad form^87^ using a quantum optics toolbox in MATLAB^55^ or PYTHON^56^. The master equation for the spin-cavity system can be written as


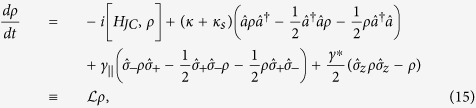


where the parameters 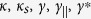 are defined in the same way as in Eq. (11), 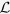 is the Liouvillian and *H*_*JC*_ is the driven Jaynes - Cummings Hamiltonian with the input field driving the cavity. In the rotating frame at the frequency of the input field, *H*_*JC*_ can be written as


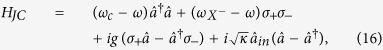


where the input field is associated with the output field and the cavity field by the input-output relation^88^, 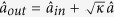.

Although an analytical solution to the master equation in Eq. (15) is very difficult, the quantum optics toolbox^55^,^56^ provides a numerical calculation of the density matrix *ρ(t*). By taking the operator average in the input-output relation, the steady-state reflection coefficient can be calculated using the following expression


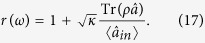


## Additional Information

**How to cite this article:** Hu, C. Y. Photonic transistor and router using a single quantum-dot-confined spin in a single-sided optical microcavity. *Sci. Rep.* **7**, 45582; doi: 10.1038/srep45582 (2017).

**Publisher's note:** Springer Nature remains neutral with regard to jurisdictional claims in published maps and institutional affiliations.
